# Afatinib circumvents multidrug resistance via dually inhibiting ATP binding cassette subfamily G member 2 *in vitro* and *in vivo*

**DOI:** 10.18632/oncotarget.2647

**Published:** 2014-11-24

**Authors:** Xiao-kun Wang, Kenneth Kin Wah To, Li-yan Huang, Jing-hong Xu, Ke Yang, Fang Wang, Zhen-cong Huang, Sheng Ye, Li-wu Fu

**Affiliations:** ^1^ State Key Laboratory of Oncology in South China, Cancer Center, Sun Yat-sen University, Collaborative Innovation Center for Cancer Medicine, Guangzhou 510060, China; ^2^ School of Pharmacy, The Chinese University of Hong Kong, Hong Kong, P.R. China; ^3^ First Affiliated Hospital, Sun Yat-sen University, Guangzhou 510080, China

**Keywords:** Multidrug resistance, ABCG2, tyrosine kinase inhibitor, afatinib, combined chemotherapy

## Abstract

Multidrug resistance (MDR) to chemotherapeutic drugs is a formidable barrier to the success of cancer chemotherapy. Expressions of ATP-binding cassette (ABC) transporters contribute to clinical MDR phenotype. In this study, we found that afatinib, a small molecule tyrosine kinase inhibitor (TKI) targeting EGFR, HER-2 and HER-4, reversed the chemoresistance mediated by ABCG2 *in vitro*, but had no effect on that mediated by multidrug resistance protein ABCB1 and ABCC1. In addition, afatinib, in combination with topotecan, significantly inhibited the growth of ABCG2-overexpressing cell xenograft tumors *in vivo.* Mechanistic investigations exhibited that afatinib significantly inhibited ATPase activity of ABCG2 and downregulated expression level of ABCG2, which resulted in the suppression of efflux activity of ABCG2 in parallel to the increase of intracellular accumulation of ABCG2 substrate anticancer agents. Taken together, our findings may provide a new and useful combinational therapeutic strategy of afatinib with chemotherapeutical drug for the patients with ABCG2 overexpressing cancer cells.

## INTRODUCTION

Intrinsic and acquired multidrug resistance (MDR) to chemotherapeutic drugs is a main obstacle for the successful cancer chemotherapy. Intense research on the mechanism of MDR has focused on the overexpression of the superfamily of ATP-binding cassette (ABC) transporters that function as active drug efflux pumps resulting in the reduction of cellular accumulation of drugs [1]. It is well established that ABCB1 (P-glycoprotein, MDR1), ABCC1 (multidrug resistance associated protein 1, MRP1) and ABCG2 (breast cancer resistance protein, BCRP) are involved in the active extrusion of anticancer drugs from cells [2]. There is emerging evidence that the expression of ABCG2 is associated with a poor clinical response to chemotherapy [3–6]. Of interest is the observation that ABCG2 has been considered as a determinant of side population (SP) cells which are highly enriched in cancer stem cells (CSCs), and appears to play a critical role in the resistance of CSCs [7, 8]. Inhibition or down-regulation of ABCG2 may be a valid approach to reverse ABCG2-mediated drug resistance and to improve the clinical efficacy of cancer chemotherapy.

Generally, the more commonly adopted approach to overcome MDR is to identify or develop effective and safe inhibitors of ABC transporters. Compared with other drug transporter inhibitors, a unique advantage of specific ABCG2 inhibitors is the putative role in the elimination of CSCs. Unfortunately, the majority of tested MDR modulators are failed because of either insufficient in efficacy or exhibiting unacceptable toxicity or unpredictable pharmacokinetic interactions [9, 10]. Recently, it is reported that ABCG2 has a relatively high affinity with some tyrosine kinase inhibitors (TKIs) which are designed to act by competing against ATP binding to the intracellular catalytic domain of oncogenic tyrosine kinases, thereby inhibiting cell growth. These TKIs, such as lapatinib and imatinib, have been shown to modulate ABC transporter activity and improve the efficacy of anticancer drugs [11–14]. Accordingly, identifying an effective TKI that can specifically inhibit or downregulate ABCG2 would dramatically accelerate the development of reversal agents for circumventing ABCG2-mediated MDR in cancer chemotherapy.

Afatinib (BIBW 2992), an ATP-competitive aniline-quinazoline compound with a reactive acrylamide group, is an orally administered irreversible inhibitor of both the epidermal growth factor receptor (EGFR) and human epidermal receptor 2 (HER2) tyrosine kinases. In June 2013, based on the good results of clinical trials, afatinib was approved by the U.S. Food and Drug Administration (FDA) for first-line treatment of patients with EGFR-mutated non small cell lung cancer (NSCLC). Afatinib is also under development in several other solid tumors including breast and head and neck cancer [15–17]. In this study, we showed that afatinib exerted inhibitory effects on ABCG2 function via dual mechanisms, competitive block of substrate transport and downregulation of ABCG2 expression, thereby reversing ABCG2-mediated drug resistance in various cancer cells with ABCG2 overexpression *in vitro* and *in vivo*.

## RESULTS

### Afatinib reversed the resistance of ABCG2-overexpressing cells to chemotherapeutic agents *in vitro*

**Figure 1:**
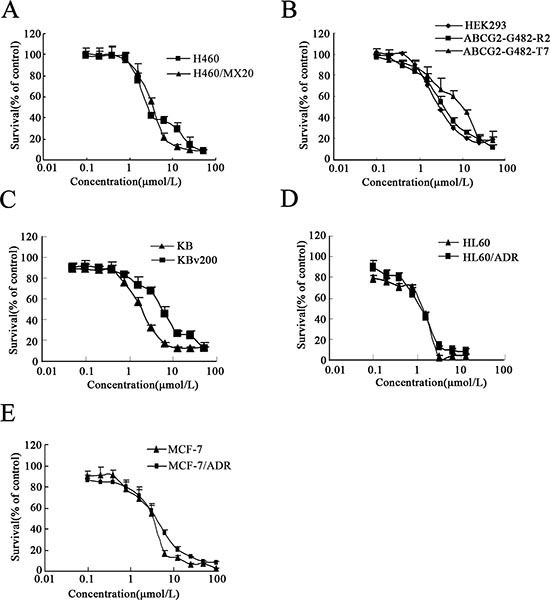
Cytotoxicity of afatinib **(A-E)** cytotoxicity of afatinib in the indicated cell lines was determined by the MTT assay. Cells were treated with varying concentrations of afatinib for 3 days. Results from three independent experiments are expressed as the Mean ± SD.

ABC transporters, especially ABCB1, ABCC1 and ABCG2, have been indicated to contribute significantly to MDR. To investigate whether afatinib could potentiate the efficacy of chemotherapeutic agents in various resistant cells, MTT assay was first used to detect the cytotoxicity of afatinib alone. As shown in Fig. 1(A-E), there was a significant difference in the susceptibility of various cells to afatinib alone. The IC_50_ values were 3.68 ± 0.09, 4.12 ± 0.06, 3.03 ± 0.06, 3.71 ± 0.13, 7.93 ± 0.12, 1.42 ± 0.10, 1.21 ± 0.09, 3.48 ± 0.28, 4.17 ± 1.48, 1.55 ± 0.38, 5.44 ± 0.14 for H460, H460/MX20, HEK293, HEK293/ABCG2-G482-R2, HEK293/ABCG2-G482-T7, HL60, HL60/ADR, MCF7, MCF7/ADR, KB and KBv200 cells, respectively. Accordingly, afatinib at concentrations of 0.1 and 1.0 μM, respectively, was selected as the maximum working concentration for further reversal assay in different cancer cells. Based on this, IC_50_ values of various drugs in different sensitive cells and in their resistant counterparts with or without the concomitant treatment with different concentrations of afatinib were shown in Table 1. The ABCG2-overexpressing cells showed significant resistant phenotype to ABCG2 substrates topotecan and mitoxantrone. Afatinib at 1.0 μmol/L significantly increased mitoxantrone-induced cytotoxicity in both the parental H460 cells and the ABCG2-overexpressing H460/MX20 cells. In addition, afatinib remarkably potentiated the efficacy of topotecan and mitoxantrone in the ABCG2-overexpressing S1-MI-80 cells, but not in the parental S1 cells which did not express ABCG2. In the presence of 1.0 μmol/L afatinib, IC_50_ values of topotecan and mitoxantrone were dramatically decreased from 7.59 ± 0.95 to 0.45 ± 0.25 μmol/L and from 15.66 ± 0.98 to 1.37 ± 0.13 μmol/L in S1-MI-80 cells, respectively (Table 1). In contrast, afatinib at 1.0 μmol/L did not significantly alter the IC_50_ values of cisplatin which is not a substrate of ABCG2, in all tested cells. These results indicated that afatinib could reverse the resistance mediated by ABCG2.

**Table 1:** Effect of afatinib on reversing ABC transporters-mediated MDR

Compounds	IC_50_ ± SD, μmol/L (Fold reversal)	
	H460	H460/MX20 (ABCG2)
Mitoxantrone	0.020 ± 0.004 (1.00)	0.369 ± 0.086 (1.00)
+0.25 μM Afatinib	0.009 ± 0.003 (2.14)	0.280 ± 0.011 (1.32)
+0.5 μM Afatinib	0.008 ± 0.002 (2.40)*	0.162 ± 0.024 (2.28)
+1.0 μM Afatinib	0.008 ± 0.002 (2.40)*	0.081 ± 0.002 (4.56)**
+2.5 μM FTC	0.007 ± 0.003 (2.86)**	0.073 ± 0.015(5.05)**
Cisplatin	2.815 ± 0.386 (1.00)	7.866 ± 0.739 (1.00)
+1.0 μM Afatinib	2.320 ± 0.248 (1.21)	8.076 ± 0.804 (0.97)
	S1	S1-MI-80 (ABCG2)
Topotecan	0.031 ± 0.006 (1.00)	7.599 ± 0.954 (1.00)
+0.25 μM Afatinib	0.028 ± 0.004 (1.11)	2.997 ± 0.504 (2.54)*
+0.5 μM Afatinib	0.023 ± 0.006 (1.35)	1.427 ± 0.215 (5.32)**
+1.0 μM Afatinib	0.022 ± 0.005 (1.43)	0.453 ± 0.252 (16.78)**
+2.5 μM FTC	0.029 ± 0.003 (1.07)	0.285 ± 0.070 (26.66)**
Cisplatin	5.546 ± 0.144 (1.00)	30.673 ± 0.988 (1.00)
+1.0 μM Afatinib	3.575 ± 0.276 (1.55)	30.119 ± 2.311 (0.98)
Mitoxantrone	0.170 ± 0.001 (1.00)	15.658 ± 0.981 (1.00)
+0.25 μM Afatinib	0.152 ± 0.002 (1.11)	7.318 ± 1.078 (2.14)*
+0.5 μM Afatinib	0.143 ± 0.002 (1.19)	4.964 ± 1.028 (3.15)*
+1.0μM Afatinib	0.164 ± 0.001 (1.04)	1.368 ± 0.131 (11.45)**
+2.5 μM FTC	0.187 ± 0.000 (0.91)	1.288 ± 0.013 (12.16)**
	KB	KBv200 (ABCB1)
Doxorubicin	0.036 ± 0.007 (1.00)	1.134 ± 0.091 (1.00)
+ 0.025 μM Afatinib	0.034 ± 0.003 (1.06)	0.835 ± 0.100 (1.36)
+0.05 μM Afatinib	0.032 ± 0.002 (1.13)	0.715 ± 0.066 (1.59)
+0.1 μM Afatinib	0.039 ± 0.002 (0.92)	0.614 ± 0.027 (1.84)
+10 μM Verapamil	0.030 ± 0.005 (1.20)	0.120 ± 0.031 (9.45)**
Paclitaxel	0.002 ± 0.003 (1.00)	0.348 ± 0.192 (1.00)
+0.025 μM Afatinib	0.019 ± 0.003 (1.05)	0.401 ± 0.012 (0.87)
+0.05 μM Afatinib	0.018 ± 0.004 (1.11)	0.302 ± 0.230 (1.15)
+0.1 μM Afatinib	0.015 ± 0.002 (1.33)	0.138 ± 0.023 (2.52)
+10 μM Verapamil	0.019 ± 0.005 (1.05)	0.051 ± 0.342 (6.82)**
Cisplatin	0.527 ± 0.998 (1.00)	0.901 ± 1.233 (1.00)
+0.1 μM Afatinib	0.589 ± 2.112 (0.89)	1.021 ± 0.772 (0.88)
	MCF-7	MCF-7/ADR(ABCB1)
Doxorubicin	0.449 ± 0.114 (1.00)	15.963 ± 1.014 (1.00)
+0.025 μM Afatinib	0.298 ± 0.184 (1.51)	10.966 ± 0.222 (1.46)
+0.05 μM Afatinib	0.368 ± 0.119 (1.22)	10.156 ± 0.612 (1.57)
+0.1 μM Afatinib	0.361 ± 0.118 (1.24)	8.462 ± 0.164 (1.88)
+10 μM Verapamil	0.401 ± 0.096 (1.12)	1.414 ± 0.078 (11.3)**
Cisplatin	5.011 ± 0.183 (1.00)	5.56 ± 1.234 (1.00)
+0.1μM Afatinib	5.883 ± 2.012 (0.84)	5.012 ± 2.061 (1.09)
	HL60	HL60/ADR(ABCC1)
Doxorubicin	0.028 ± 0.003 (1.00)	6.227 ± 0.588 (1.00)
+0.025 μM Afatinib	0.027 ± 0.001 (1.04)	4.795 ± 0.305 (1.30)
+0.05 μM Afatinib	0.034 ± 0.002 (0.82)	5.155 ± 0.543 (1.21)
+0.1 μM Afatinib	0.026 ± 0.001 (1.08)	5.521 ± 0.466 (1.13)
+0.7 μM MK571	0.024 ± 0.003 (0.86)	2.007 ± 0.023 (3.10)*
Cisplatin	1.533 ± 0.143 (1.00)	1.587 ± 1.234 (1.00)
+0.1 μM Afatinib	1.626 ± 0.917 (0.95)	1.603 ± 0.102 (0.99)

Cell survival was determined by MTT assay as described in Materials and Methods. The fold reversal of MDR (values given in parentheses) was calculated by dividing the IC_50_ for cells with anticancer drugs in the absence of afatinib by that obtained in the presence of afatinib. Data represent Mean ± SD of at least three independent experiments. *p < 0.05, **p < 0.01.

The effect of afatinib on the ABCB1 and ABCC1 transporters was also determined by MTT assay. It was found that afatinib did not enhance the cytotoxicity of doxorubicin, which is a known substrate for both ABCB1 and ABCC1, in resistant KBv200, MCF7/ADR and HL60/ADR cells that express ABCB1, ABCB1 and ABCC1, respectively, suggesting that afatinib probably did not interact with ABCB1 and ABCC1.

It has been reported that mutations in ABCG2 protein at amino acid 482 may alter the substrate specificity and undermine the effectiveness of ABCG2 inhibitor [18]. Therefore, the potentiation of cytotoxicity of ABCG2 substrate drugs by afatinib was also investigated in HEK293 cells stably transfected with wild-type (482R2) or mutant (482T7) ABCG2. These ABCG2-stably transfected HKE293 cells exhibited moderate levels of resistance to ABCG2 substrates (topotecan or mitoxantrone) compared with cells transfected with a control vector (Table 2). In HEK293 cells transfected with wild type ABCG2 (482R2) vector, at a concentration of 1.0 μmol/L, afatinib shifted the IC_50_ for topotecan and mitoxantrone from 0.233 ± 0.069 and 0.470 ± 0.008 to 0.027 ± 0.006 and 0.030 ± 0.001 μmol/L, respectively. A similar enhancement in topotecan and mitoxantrone cytotoxicity was also observed in HEK293 cells expressing the mutant ABCG2 (482T7); afatinib at 1.0 μmol/L significantly decreased IC_50_ values for topotecan and mitoxantrone, from 0.446 ± 0.036 and 0.533 ± 0.298 to 0.073 ± 0.032 and 0.094 ± 0.045 μmol/L, respectively. While the anticancer activity of topotecan or mitoxantrone was not altered in the control vector-transfected HEK293 with or without the concomitant treatment of afatinib.

**Table 2:** Effect of afatinib on reversing ABCG2-mediated MDR in transfected cell lines

Compounds	IC_50_ ± SD, μmol/L (Fold reversal)		
	HEK293/pcDNA3.1	ABCG2-482-R2	ABCG2-482-T7
Topotecan	0.030 ± 0.008 (1.00)	0.233 ± 0.069 (1.00)	0.446 ± 0.036 (1.00)
+0.25 μM Afatinib	0.025 ± 0.008 (1.20)	0.167 ± 0.032 (1.39)	0.290 ± 0.030 (1.54)
+0.5 μM Afatinib	0.023 ± 0.005 (1.31)	0.071 ± 0.016 (3.29)*	0.157 ± 0.036 (2.84)
+1.0 μM Afatinib	0.016 ± 0.004 (1.84)	0.027 ± 0.006 (8.64)*	0.073 ± 0.032 (6.10)*
+2.5 μM FTC	0.009 ± 0.090 (3.33)*	0.013 ± 0.031 (17.9)*	0.058 ± 0.018 (7.69)*
Cisplatin	5.934 ± 0.268 (1.00)	6.598 ± 0.037 (1.00)	3.620 ± 0.036 (1.00)
+1.0 μM Afatinib	3.935 ± 0.146 (1.51)	8.695 ± 0.034 (0.76)	3.778 ± 0.032 (0.96)
Mitoxantrone	0.070 ± 0.001 (1.00)	0.470 ± 0.008 (1.00)	0.533 ± 0.298 (1.00)
+0.25 μM Afatinib	0.060 ± 0.001 (1.21)	0.480 ± 0.003 (0.98)	0.359 ± 0.175 (1.49)
+0.5 μM Afatinib	0.040 ± 0.001 (1.87)	0.190 ± 0.002 (2.43)	0.172 ± 0.140 (3.09)*
+1.0 μM Afatinib	0.030 ± 0.000 (2.33)	0.042 ± 0.018 (11.20)*	0.094 ± 0.045 (5.67)*
+2.5 μM FTC	0.030 ± 0.001 (2.33)	0.030 ± 0.001 (13.75)*	0.035 ± 0.029 (15.07)*

Cell survival was determined by MTT assay as described in Materials and Methods. The fold reversal of MDR (values given in parentheses) was calculated by dividing the IC_50_ for cells with anticancer drugs in the absence of afatinib by that obtained in the presence of afatinib. Data represent Mean ± SD of at least three independent experiments. *p < 0.01.

Taken together, these results indicated that afatinib sensitized ABCG2-overexpressing cells to chemotherapeutic agents *in vitro*.

### Afatinib significantly potentiated the anticancer activity of topotecan *in vivo*

**Figure 2:**
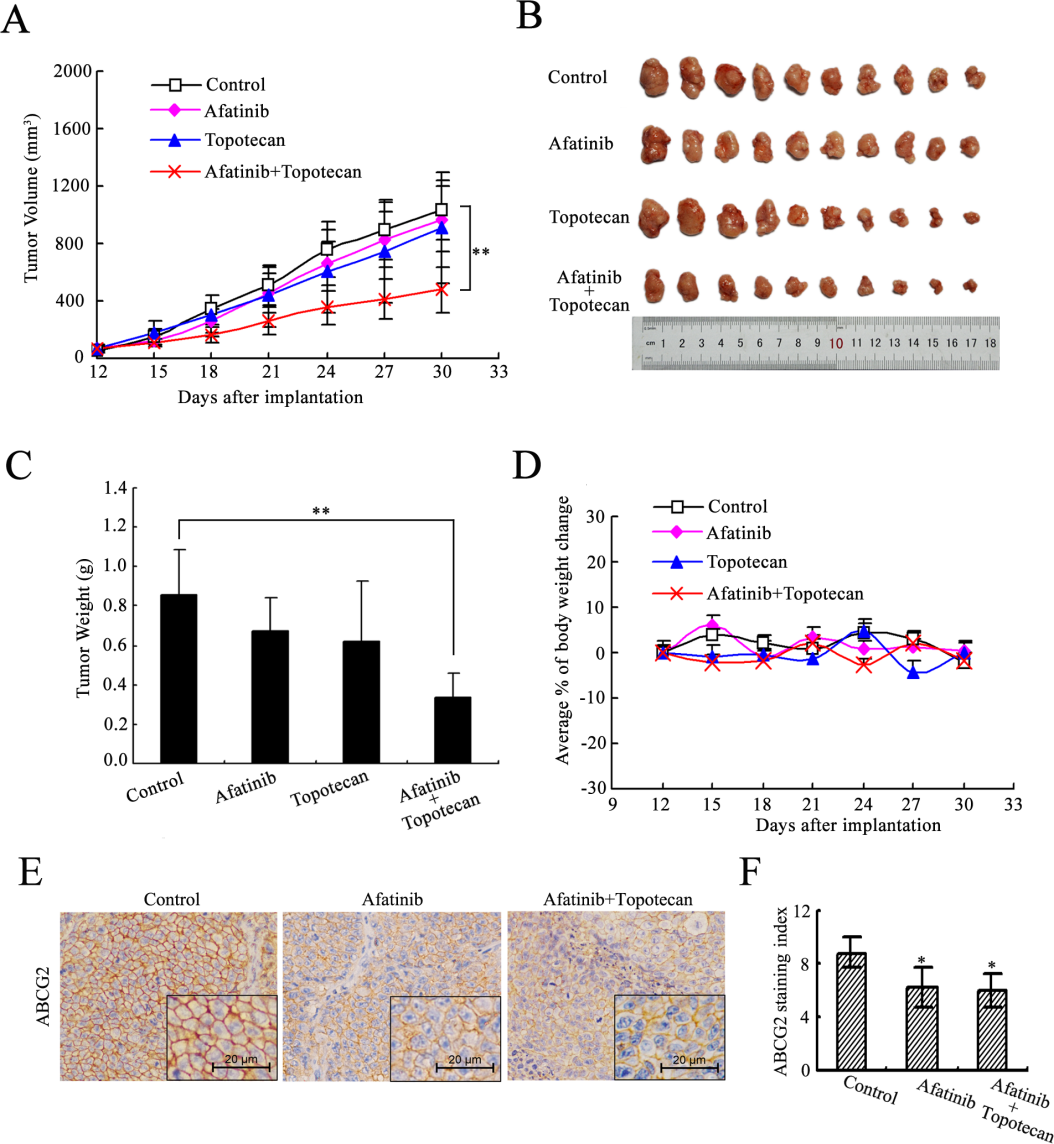
Afatinib potentiated the anticancer activity of topotecan in the H460/MX20 cell xenograft tumors Athymic nude mice with xenograft tumors by injecting subcutaneously H460/MX20 cells (3 × 10^6^) were treated with saline, afatinib, topotecan, or the combination of afatinib with topotecan, respectively, over a period of 18 days. Tumor growth was monitored every 3 days. **(A)** the changes in tumor volume over time following the implantation. Data points represent the mean ± SD of tumor volumes from each group. *n* = 12. **(B)** image of excised xenograft tumors from four groups. **(C)** mean tumor weight (*n* = 12) of excised xenograft tumors. Error bars indicate SD. **(D)** the changes in body weight. Each point represents the mean ± SD of body weight from each group. *n* = 12. **(E)** immunohistochemical staining of xenograft tumors for ABCG2. Representative sections obtained from paraffin-embedded H460/MX20 xenograft tumors were immunostained for expression of ABCG2. **(F)** xenograft tumors treated with afatinib alone or combination with topotecan showed a reduced ABCG2 expression compared with saline-treated tumors (Wilcoxon test). ABCG2 staining index means percent of positively stained tumor cells×staining intensity (0, 1, 2 and 3).*p < 0.05, **p < 0.01. versus the saline group.

H460/MX20 cell xenograft tumor model was established to investigate the sensitizing effect of afatinib *in vivo*. As shown in Fig. 2(A-B), neither afatinib nor topotecan alone produced significant antitumor effect. In contrast, afatinib in combination with topotecan significantly decreased the sizes, weights and volumes of xenograft tumors, compared with saline, afatinib or topotecan alone. The mean weights of tumors excised from mice were 0.86 ± 0.23, 0.67 ± 0.17, 0.62 ± 0.31, 0.34 ± 0.12g for saline, afatinib, topotecan and combination groups, respectively (Fig. 2C). And the inhibition rate (IR) of the combination group was up to 60.43 %. Throughout the *in vivo* study, afatinib alone or combination group did not produce visible weight loss or treatment-related deaths in the athymic nude mice (Fig. 2D). These results indicated that the antitumor ability of topotecan was significantly enhanced when it was administrated in combination with afatinib in the tumors that expressed ABCG2.

Furthermore, we used these xenograft tumors to test the effect of afatinib on ABCG2 expression *in vivo* by performing ABCG2 immunohistochemical staining. H460/MX20 xenograft tumors exhibited an intense positive staining for ABCG2 on the cell surface (Fig. 2E). Xenograft tumors of saline control group showed higher ABCG2 staining compared with tumors that treated with afatinib alone or combination with topotecan (Fig. 2F). These findings suggest that the enhanced anticancer activity of topotecan by afatinib *in vivo* might be due to impaired ABCG2 expression.

### Afatinib inhibited efflux activity of ABCG2

**Figure 3:**
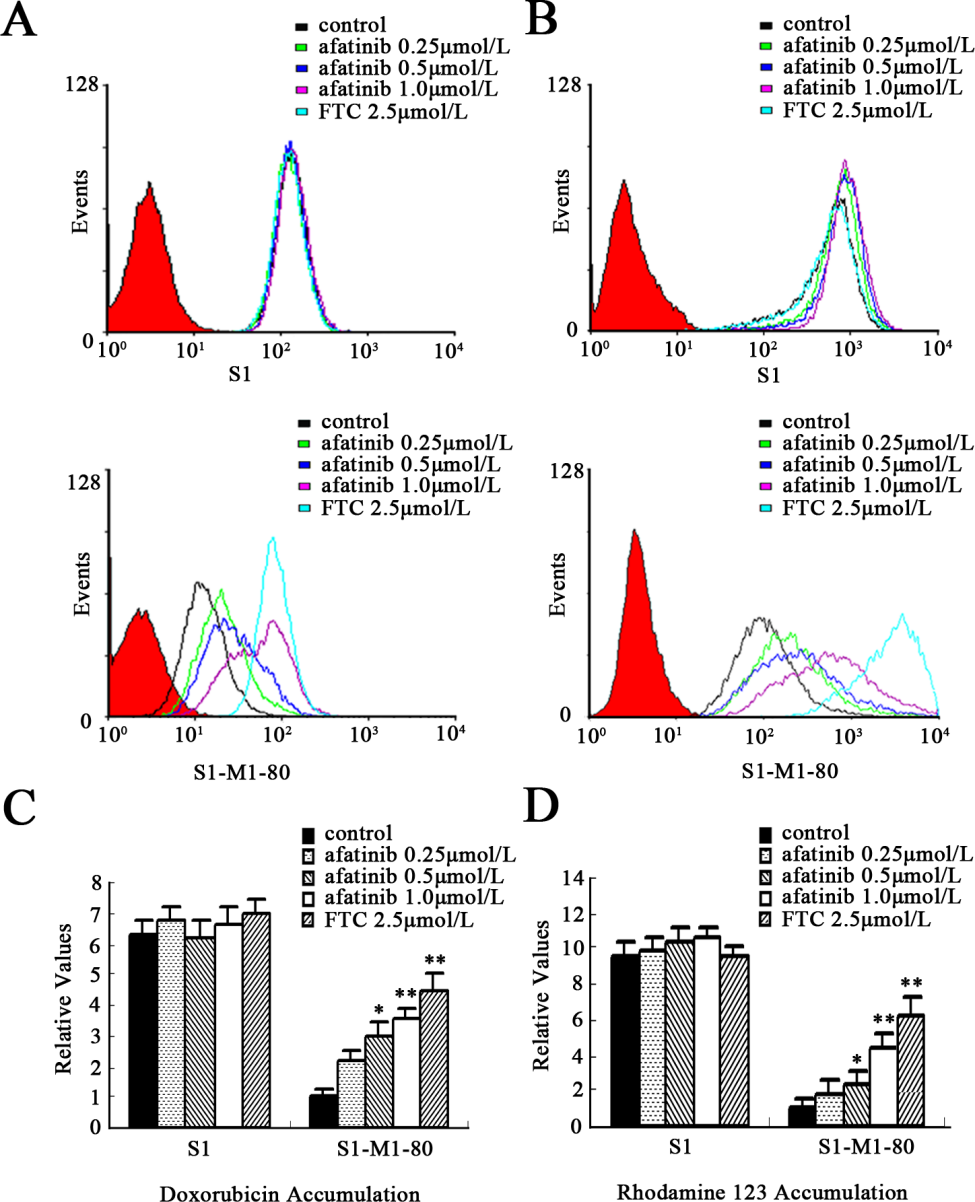
Effect of afatinib on the intracellular accumulation of Dox and Rho123 in S1 and S1-MI-80 cells The accumulation of Dox **(A, C)** and Rho 123 **(B, D)** was measured by flow cytometry as described in Materials and Methods. All of these experiments were repeated at least thrice. Data represent Mean ± SD. *p < 0.05, **p < 0.01.

The potentiation of anticancer activity by transporter inhibitors is usually mediated by the inhibition of transporter-mediated efflux, thereby leading to an increase in the intracellular drug accumulation [19]. To explore the potential mechanism by which afatinib sensitizes ABCG2-overexpressing cells to chemotherapeutic drugs, we examined the intracellular accumulation of doxorubicin (Dox) and Rho 123, known fluorescent substrates of ABCG2, by flow cytometry in S1-MI-80 cells. As shown in Fig. 3(A-B), the intracellular concentrations of Dox and Rho 123 in S1-MI-80 cells were significantly lower than that in their parental S1 cells in the absence of afatinib. But in the presence of 0.25, 0.5 or 1.0 μmol/L afatinib, the fluorescence index of Dox in S1-MI-80 cells was elevated by 2.2-, 3.0-, 3.5-fold, respectively (Fig. 3C). The intracellular accumulation of Rho123 was increased by 1.7-, 2.2- and 4.5-fold, respectively (Fig. 3D). These results suggest that afatinib, similar to a potent ABCG2-specific inhibitor FTC, dramatically increased the accumulation of Dox and Rho 123 in a concentration-dependent manner in S1-MI-80 cells (Fig. 3). However, neither afatinib nor FTC affected the intracellular levels of Dox and Rho123 in S1 cells.

**Figure 4:**
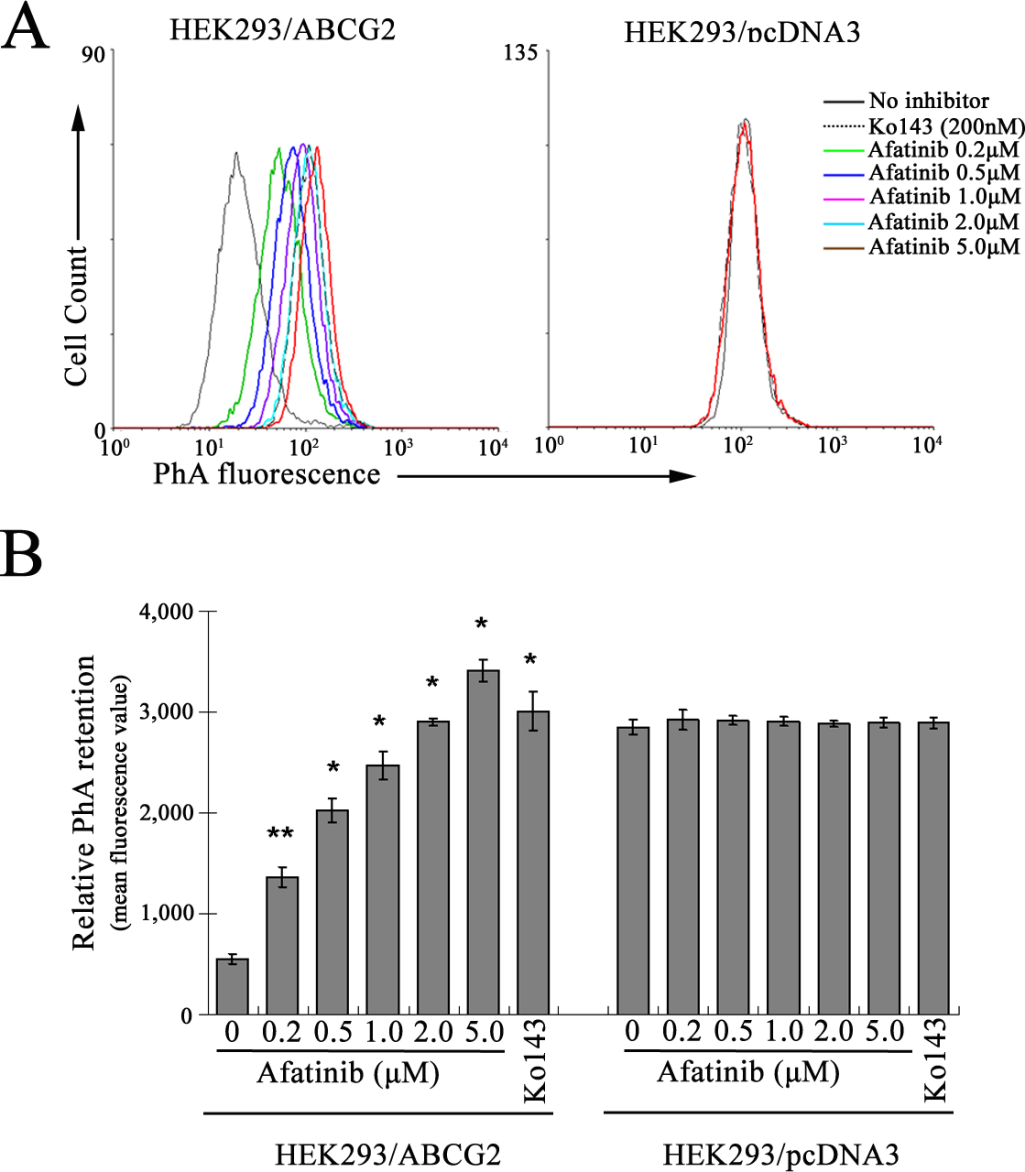
Inhibition of ABCG2-mediated PhA efflux by afatinib HEK293/ABCG2 or pcDNA3 cells were incubated with 1 μM PhA alone (black solid line), 1 μM PhA with 200 nM Ko143 (dotted line), or 1 μM PhA with afatinib at the indicated concentrations at 37°C for 30 min. PhA fluorescence retention in the cells after a 1-h PhA-free efflux was measured by flow cytometry. Representative histograms from three independent experiments are shown in **(A)**. For clarity, only efflux histograms corresponding to PhA with or without 200 nM Ko143 and 20 μM afatinib are shown for the HEK293/pcDNA3 cells. **(B)** relative PhA retention in the cells is expressed as mean fluorescence value to summarize the ABCG2 efflux inhibitory effect of afatinib. Columns, means of triplicate determinations; bars, SD. *p < 0.01, versus HEK293/pcDNA3 cells unincubated with Ko143.

In addition, the competition between afatinib and a fluorescent ABCG2 probe substrate (pheophorbide A, PhA) for efflux was studied in HEK293/ABCG2 cells by flow cytometry analysis. The read-out of the assay is the retention of the fluorescent ABCG2 substrate (PhA) after a 1-h drug-free efflux. Inhibition of ABCG2-mediated efflux is indicated by a shift to higher intracellular fluorescent signal. As illustrated in Fig. 4(A-B), afatinib was found to inhibit the efflux of PhA in a concentration-dependent manner. Compared with another specific and potent ABCG2 inhibitor Ko143, afatinib at a concentration of 2 μM exhibited similar inhibitory effect on ABCG2-mediated efflux as 200 nM Ko143. The inhibition may be specific because intracellular fluorescence in the backbone vector-transfected HEK293/pcDNA3 cells was not affected by afatinib (Fig. 4A-B).

### Increased 5D3 labeling by afatinib suggest its interaction with ABCG2

**Figure 5:**
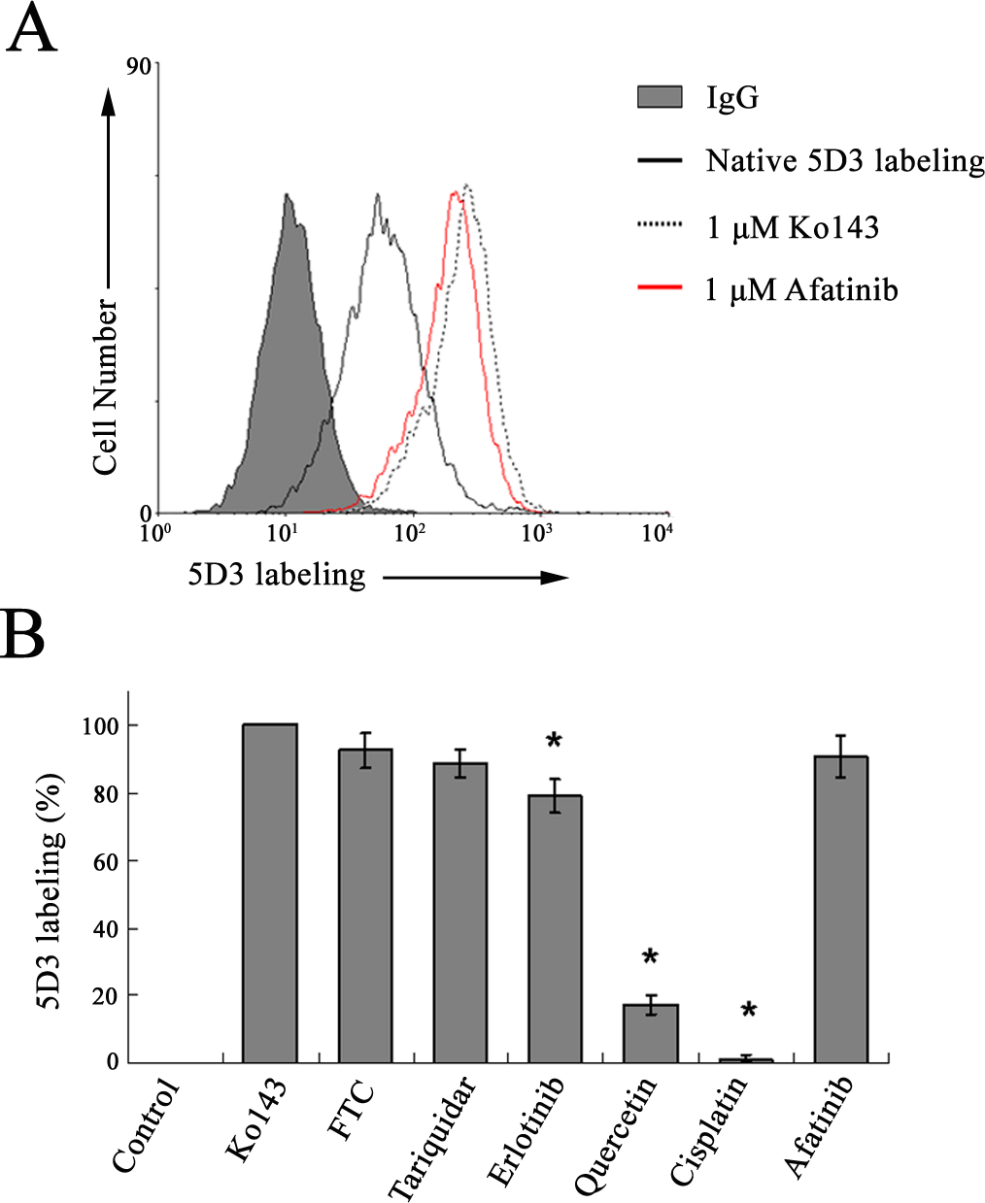
5D3 labeling in ABCG2-stably transfected HEK293 cells, suggesting interaction between afatinib and ABCG2 **(A)** representative 5D3 shift histogram exhibited by Ko143 (control specific ABCG2 inhibitor) and afatinib. The solid line represents 5D3 binding of the untreated (native) cells and the dotted line for the cells incubated with 1 μM Ko143 (red line for 1 μM afatinib). The shaded histogram represents the background fluorescent signal upon labeling with a normal mouse IgG2b (isotype control). **(B)** comparison of 5D3 shift produced by afatinib and other known ABCG2 inhibitors/substrates. Known ABCG2 inhibitors: Ko143 (1 μM), FTC (5 μM), tariquidar (1 μM) and erlotinib (10 μM); known ABCG2 substrate: quercetin (25 μM); reported non-ABCG2 substrate: cisplatin (50 μM). The various tested compounds were present during the 45 min antibody labeling. Fluorescence values are shown as the percentage of maximum labeling obtained in HEK293 ABCG2 cells incubated with 1 μM Ko143 (set as 100%) and labeled with 5D3. Mean and SD of the mean channel numbers from histograms obtained from three independent experiments is plotted. *p < 0.01, versus the 5D3 shift mediated by Ko143.

5D3 is a conformation sensitive monoclonal antibody recognizing an extacellular epitope of the human ABCG2. 5D3 binding to ABCG2 was known to be increased in certain conformations of the transporter protein upon substrate/inhibitor binding and ATP hydrolysis (i.e. 5D3 shift) [20]. The 5D3 shift assay was therefore performed in HEK293 ABCG2 cells to demonstrate the interaction of afatinib with ABCG2. Using the specific ABCG2 inhibitor (Ko143, 1 μM) as the positive control (set as 100% 5D3 labeling for comparison) (Fig. 5A), afatinib (1 μM) was found to produce a remarkable 5D3 shift close to the level attained by Ko143, thus suggesting the interaction between afatinib and ABCG2. Other known ABCG2 inhibitors (including FTC, tariquidar and erlotinib) tested were also shown to notably increase 5D3 labeling relative to the untreated control (Fig. 5B). On the other hand, quercetin (a known ABCG2 substrate) was found to increase only slightly the 5D3 shift (~20% that of Ko143) whereas cisplatin (a non-ABCG2 substrate) did not appreciably affect 5D3 labeling.

### ATPase activity of ABCG2 was inhibited by afatinib in a dose dependent manner

**Figure 6:**
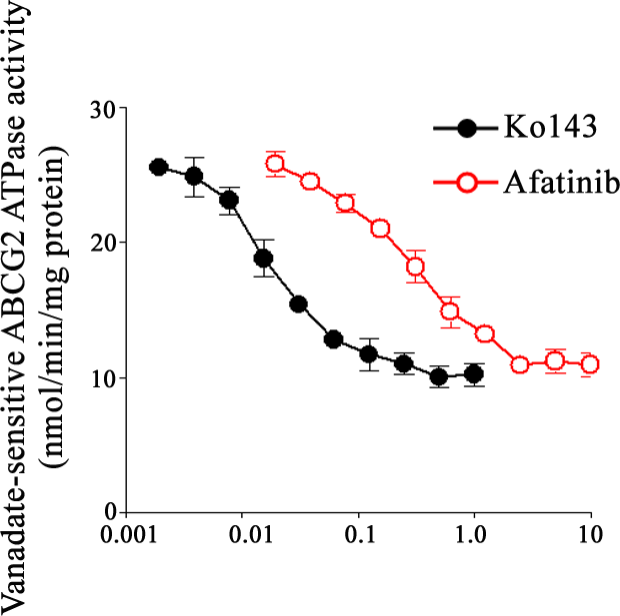
Effect of afatinib on the ATPase activity of ABCG2 The vanadate-sensitive ATPase activity of ABCG2 in crude membranes isolated frm ABCG2-expressing High Five insect cells was determined over a range of different concentration of afatinib. ATP hydrolysis was monitored by measuring the amount of inorganic phosphate released using a colorimetric assay. Ko143 (specific ABCG2 inhibitor) was also tested as control for comparison. Mean of triplicate measurements is presented (bars, SD).

Drug efflux function of ABCG2 is associated with ATP hydrolysis that is modulated in the presence of its substrates or inhibitors. To further understand the mechanism of ABCG2 inhibition by afatinib, the ATPase activity of ABCG2 was measured in the presence of a range of concentrations of afatinib. Sodium orthovanadate was used in this experiment to suppress other major membrane ATPases during the measurement. Similar to the specific ABCG2 inhibitor (Ko143), afatinib was found to inhibit the ATPase activity of ABCG2 in a dose dependent manner, albeit its inhibitory effect was less potent than that of Ko143 (Fig. 6).

### Afatinib downregulated ABCG2 expression

**Figure 7:**
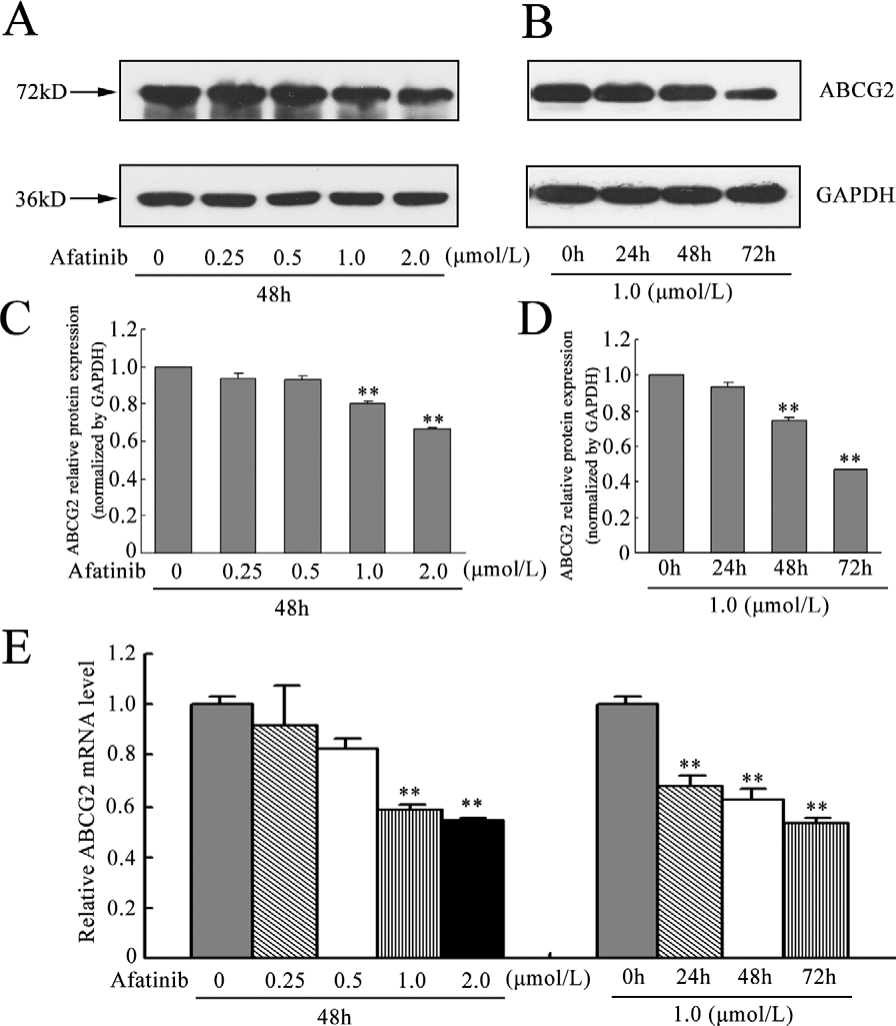
Effect of afatinib on the expression of ABCG2 **(A-B)** H460/MX20 cells were treated with varying concentrations (0–2.0 μM) of afatinib for 48 h, or with 1.0 μM afatinib for 24 h, 48 h and 72 h, respectively. ABCG2 protein levels were analyzed by Western blot. GAPDH was used as a loading control. **(C-D)** relative quantification of the effect of afatinib on ABCG2. ABCG2 protein expression levels were normalized to GAPDH. **(E-F)** effect of afatinib on the ABCG2 expression at the mRNA level was determined by real time RT-PCR. The amount of ABCG2 mRNA in a given sample was normalized to the level of GAPDH in that sample. The 2^−ΔΔCt^ method was used to analyze the relative change. Data represent Mean ± SD. **p < 0.01.

To evaluate the effect of afatinib on the expression of the drug transporter ABCG2, we determined the expression of ABCG2 at protein and mRNA level in H460/MX20 cells after exposure to varying concentrations of afatinib. It was found that afatinib reduced ABCG2 protein level although the reduction was not significant at relatively low concentrations (Fig. 7A-B). To further determine this, we incubated the cells with 1.0 μM afatinib for 0, 24, 48, 72 h, respectively. The expression levels of ABCG2 were found to decrease after exposure to afatinib in H460/MX20 cells, thus confirming the immunohistochemical staining results that afatinib reduced ABCG2 expression (Fig. 7C-D). Furthermore, in the presence of afatinib, the alteration of ABCG2 mRNA expression level was consistent with that of protein level, suggesting that afatinib probably downregulate ABCG2 expression at the transcriptional level (Fig. 7E).

All these findings showed that the downregulation of ABCG2 may contribute to the sensitizing effect by afatinib in the ABCG2-overexpressing cells.

## DISCUSSION

Multidrug resistance (MDR) of tumor cells to chemotherapeutic agents is known to be the main cause for treatment failure in cancer chemotherapy. Energy-dependent efflux of chemotherapeutics by several ABC transporters, mainly by ABCB1, ABCC1 and ABCG2, has been reported as important contributing factor to the development of MDR [21, 22]. Current strategies against MDR are intended to reverse or prevent resistance of cancer cells, by administering a transporter inhibitor at the beginning of chemotherapy or using anticancer drugs that are not substrates of drug transporters. Besides, a newly proposed strategy for treating drug resistant cancers is to exploit the resistance of cancer cells, which was achieved by administrating at least two agents in sequence to selectively protect non-resistant cells with killing of drug-resistant cancer cells [23–25]. More recently, one potential finding is that the combination of TKIs and cytotoxic anticancer drugs is likely to have an additional beneficial effect [13, 26, 27]. A hot area of translational research that requires more *in vivo* studies is the use of TKIs as agents that can potentially modify the functional activity of ABC transporters [12, 28]. Therefore, TKIs can be exploited to overcome resistance by increasing the intracellular concentration of P-gp and/or ABCG2 substrate anticancer drugs in cancer cells, tissues or tumors that express high levels of these transporters [29].

Afatinib, a new FDA-approved TKI, is well known for its effectiveness against advanced or metastasis non-small cell lung cancer with mutant EGFR. We hypothesized that afatinib can effectively compete with chemotherapeutic agents for binding with ABCB1, ABCC1, or ABCG2 and thus increase drug concentrations in resistant cancer cells. But in this study, one potential finding is that afatinib could increase the sensitivity of ABCG2-overexpressing cells to chemotherapeutic agents which are substrates of ABCG2 in a dose-dependent manner, but did not potentiate the cytotoxicity of cisplatin, a drug that is not the substrate for ABCG2 transporter. In addition, in various sensitive parental cell lines, there was no additive or synergetic antitumor effect. In contrast, in resistant cancer cells with ABCB1/ABCC1 overexpression, afatinib can not enhance the cytotoxicity of doxorubicin, a substrate drug for both ABCB1 and ABCC1, suggesting that afatinib probably did not interact with ABCB1 or ABCC1. Under all the experimental concentrations, afatinib itself had no cytotoxic effect on the cancer cells. Based on our *in vitro* study, the antitumor effect of combination of afatinib with topotecan was also demonstrated in a H460/MX20 cell xenograft model. The results indicated that combination of afatinib with topotecan exerted a better antitumor efficacy. Compared with saline group, the inhibition rate of the combination group was elevated by 60.43%. All these data indicated that afatinib could act as a potent inhibitor of ABCG2 to reverse the multidrug resistance medicated by ABCG2 *in vitro* and *in vivo*.

The 5D3 shift assay suggested the interaction of afatinib with ABCG2. Drug accumulation and efflux assay by flow cytometry revealed that afatinib inhibited the efflux capacity of ABCG2 in a dose-dependent manner. Therefore, the ability of afatinib to reverse MDR mediated by ABCG2 may be explained by its inhibitory effect on the efflux of ABCG2. Drug efflux function of ABCG2 is associated with ATP hydrolysis that is modulated in the presence of its substrates or inhibitors. To further understand the mechanism underlying ABCG2 inhibition by afatinib, the ATPase activity and expression level of ABCG2 was measured in the presence of a range of concentrations of afatinib. Notably, afatinib was found to inhibit the ATPase activity of ABCG2 in a dose dependent manner. Moreover, afatinib partially suppressed the expression of ABCG2 at both the protein and mRNA level at the relatively high concentrations. Thus, afatinib probably exerted inhibitory effects on ABCG2 via dual mechanisms, competitive block of substrate transport and downregulation of ABCG2 expression. Further studies are still needed to elucidate the exact mechanism underlying the effect of afatinib on ABCG2 expression.

Recently, ABCG2 has been proposed to be a promising biomarker for the identification and a new therapeutic target for the eradication of CSCs [30]. Also, ABCG2 is responsible for the “side population” (SP) phenotype, which is often used for the identification and isolation of CSCs. Currently, conventional chemotherapeutic agents kill primarily the highly proliferative tumor cells. The CSCs are spared by their high expression of multidrug transporters (especially, ABCG2), mediating their chemoresistance, and eventually lead to tumor relapse and metastasis. Completely eliminating cancer stem cells by overcoming the resistance to chemotherapy mediated by ABCG2 would be a new therapeutic target. Although it has been reported that various TKIs including the reversible EGFR inhibitors gefitinib and erlotinib could inhibit ABCG2, the inhibitory effect was generally achieved by directly blocking the drug efflux activity of ABCG2, with no change in its expression. In contrast, in this study, we showed that afatinib could reverse the drug resistance and enhance the cytotoxicity of conventional anticancer drugs in ABCG2-overexpressing cancer cells by a dual inhibition of ABCG2. In addition to its ability to inhibit ABCG2 activity, afatinib also reduced the expression of ABCG2, albeit relatively moderate. This mode of action of afatinib on ABCG2 made it distinctive from other TKIs and clearly increased the potency of afatinib on ABCG2. Importantly, ABCG2 has been reported to be a predictor of shorter survival in cancer patients, which implied that inhibiting ABCG2 might contribute to increased response and prolonged survival rates [31]. Icotinib, a new small- molecule inhibitor of EGFR tyrosine kinase, has been showed to interact with ABCG2 transporter and reverse ABCG2-mediated MDR by antagonizing the drug efflux function of ABCG2, with no effect on ABCG2 expression [32]. It is unknown what fundamental differences between afatinib and other TKIs such as icotinib cause the difference in their mechanism of action on ABCG2, which need to be evaluated in future in-depth studies. Regardless, this study suggested that afatinib may be a better candidate of ABCG2 inhibitor. Combined chemotherapy of afatinib with topotecan may provide a more effective way of sensitizing ABCG2-mediated MDR and probably eliminating CSCs.

In summary, this study showed that afatinib could reverse the drug resistance and enhance the cytotoxicity of conventional anticancer drugs in ABCG2-overexpressing cancer cells by a dual inhibition of ABCG2: inhibiting the drug transport function and downregulating the expression of ABCG2. These findings suggest that afatinib may be a good candidate of ABCG2 inhibitors, and could be used for combined chemotherapy with conventional anticancer drugs to achieve a better therapeutic effect, which merits further clinical investigation.

## METHODS

### Chemicals and reagents

Mitoxantrone, topotecan, doxorubicin, paclitaxel, rhodamine123, verapamil, and fumitremorgin C (FTC) were purchased from Sigma Chemical Co. (St. Louis, USA). Afatinib (BIBW2992), whose molecular structure was shown in Figure 1A, was purchased from Med Chem Express Co. (USA) and dissolved in DMSO for use at indicated concentrations.

### Cell culture

Human non-small cell lung carcinoma cell line NCI-H460 and mitoxantrone- selected ABCG2-overexpressing subline NCI-H460/MX20, human colon carcinoma cell line S1 and mitoxantrone-selected ABCG2-overexpressing subline S1-MI-80, human leukemia cell line HL60 and its doxorubicin-selected ABCC1-overexpressing derivative cell line HL60/ADR, human breast carcinoma cell line MCF-7 and the doxorubicin-resistant, ABCB1-overexpressing cell line MCF-7/ADR, stably transfected HEK293/pcDNA3.1, ABCG2-482-R2 (wild-type) and ABCG2-482-T7 cells carrying either an empty pcDNA3.1 vector or a pcDNA3.1 vector containing full-length ABCG2 coding either arginine (R) or threonine (T) at the amino acid 482 position, respectively [18], were kindly provided by Dr. Susan Bates (National Institutes of Health, Bethesda, MD). The human oral epidermoid carcinoma cell line KB and its vincristine-selected derivative ABCB1-overexpressing cell line KBv200 were generous gifts from Dr. Xu-Yi Liu (Cancer Hospital of Beijing, Beijing, China). All cell lines were cultured in RPMI1640/DMEM supplemented with 10% fetal bovine serum, 100 U/mL penicillin and 100 U/mL streptomycin at 37°C in 5% (v/v) CO_2_.

### Cytotoxicity assay

Cytotoxicity was determined using MTT assay as follows. Cells were seeded in 96-well plates at the appropriate density. After plating for 24 h, cells were treated with increasing concentrations of afatinib for another 68 h at 37°C. Then, MTT (5 mg/mL) was added into the cells and incubated for another 4 h. Then the medium was removed followed by adding 200 μL of dimethylsulfoxide (DMSO). Cytotoxicity was assessed by use of the Model 550 Microplate Reader (BIO-RAD, Hercules, CA, USA). Both the fitted sigmoidal dose response curve and IC_50_ were calculated by use of the Bliss method [33].

The reversal experiments *in vitro* were carried out as previously described [34]. The fold of resistance was calculated by dividing the IC_50_ for the MDR cells by that for the parental sensitive cells. The degree of reversal of MDR (fold reversal) was calculated by dividing the IC_50_ for cells with the anticancer drug in the absence of afatinib by that obtained in the presence of afatinib.

### Animal experiments

*In vivo* experiments were done in accordance with the guidelines for the use of laboratory animals of the Sun Yat-Sen University Institutional Animal Care and Use Committee. H460/MX20 cells (3 × 10^6^) were subcutaneously injected into the right flank of athymic nude mice (BALB/c-nu/nu, both sexes, 5 to 6 weeks old). When xenograft size reached 5 mm in diameter, mice were randomized into four groups (12 in each group), and then received various treatments: (a) saline (every 3 d × 6, intraperitoneally [IP]); (b) topotecan (every 3 d × 6, IP, 3 mg/kg); (c) afatinib (every 3 d × 6, orally [PO], 20 mg/kg); (d) topotecan (every 3 d × 6, IP, 3 mg/kg) plus afatinib (every 3 d × 6, PO, 20 mg/kg) (afatinib was given 1 h before topotecan administration). Tumor size was measured with linear calipers every 3 days. Tumor volumes (V) were calculated using the formula: (length×width^2^/2). The mice were euthanized on day 30 and the xenografts were excised and weighed. The ratio of growth inhibition (IR) was estimated according to the following formula:

$$\text{IR(\%)=}\frac{1-\text{Mean tumor weight of experimental group}}{\text{Mean tumor weight of control group}}\times 100$$

### Intracellular drug accumulation assay

The intracellular accumulation assay of Dox and rhodamine123 was performed as previously described with minor modifications [35]. Briefly, cells in culture were preincubated with various concentrations of afatinib, FTC (as a positive control inhibitor of ABCG2) or vehicle control for 3h at 37°C, followed by addition of 10μmol/L Dox or 5 μmol/L rhodamine123 and incubation for an additional 3 h or 0.5 h, respectively. The cells were then collected, centrifuged and washed three times with ice-cold PBS. Finally, the cells were analyzed with flow cytometry (Cytomics FC500, Beckman Coulter). Dox and rhodamine123 fluorescence were detected with a 488-nm argon laser, and a 515-nm bandpass filter and a 575-nm bandpass filter, respectively.

### Flow cytometry-based ABCG2 substrate efflux assay

A flow cytometry-based assay was employed to study the inhibition of ABCG2 transport function by afatinib as described previously [36]. Briefly, HEK293 cells stably transfected with ABCG2 or the backbone vector pcDNA3 (i.e. HEK293/ABCG2 and pcDNA3, respectively) were trypsinized and incubated for 30 min in phenol red-free complete medium with 1 μM pheophorbide A (PhA) in the presence or absence of a range of different concentrations of afatinib. Subsequently, the cells were washed twice with ice-cold PBS and incubated in PhA-free medium for 1 h at 37°C continuing with the tested inhibitor to generate the inhibitor/efflux histogram, or without the inhibitor to generate the efflux histogram. The inhibited efflux was determined as the difference in mean fluorescence value. To determine significant difference between intracellular fluorescence values, the Student's t-test was performed with *p* < 0.05 being considered significant. Cells were finally washed with cold Dulbecco's PBS and placed on ice in the dark until analysis by flow cytometry. Ko143, a specific ABCG2 inhibitor, was used as the control for comparison. Samples were analyzed on a LSRFortessa Cell Analyzer (BD Biosciences, San Jose, CA). PhA fluorescence was detected with a 488-nm argon laser and a 670-nm bandpass filter. At least 10,000 events were collected for all flow cytometry studies. Cell debris was eliminated by gating on forward versus side scatter and dead cells were excluded based on propidium iodide staining. All assays were performed in three independent experiments

### 5D3 shift assay for assessing interaction between afatinib and ABCG2

The binding of the conformational sensitive 5D3 antibody to ABCG2 in intact cells incubated with or without afatinib was evaluated by flow cytometry as described previously [37]. Cells were preincubated with afatinib in 0.5% bovine serum albumin/Dulbecco's PBS for 10 min at 37°C before labeling with 0.5μg/mL of either phycoerythrin-conjugated anti-ABCG2 antibody 5D3 (eBioscience, San Diego, CA) or the phycoerythrin-conjugated mouse IgG2b control antibody (eBioscience) for another 45 min at 37°C. The tested compounds were present during the antibody labeling. As positive control for maximum labeling, 5D3 binding was determined in the presence of 1μM Ko143 (a known specific ABCG2 inhibitor). Samples were analyzed on a LSRFortessa Cell Analyzer (BD Biosciences, San Jose, CA). Fluorescence was detected with a 488-nm argon laser and a 575-nm bandpass filter. At least 10,000 events were collected for all flow cytometry studies. All assays were performed in three independent experiments.

### ABCG2 ATPase assay

The vanadate-sensitive ATPase activity of ABCG2 was determined as previously described with minor modifications. Crude membranes isolated from ABCG2-expressing high five insect cells was kindly provided by Dr. Suresh Ambudkar (National Cancer Institute, NIH, USA). Briefly, afatinib (0.02–10 μM) or Ko143 (control specific ABCG2 inhibitor; 0.002–1 μM) were allowed to incubate with the crude membrane (100 μg/mL protein) in the presence or absence of 1.2 mM sodium orthovanadate in an ATPase assay buffer (50 mM KCl, 5 mM sodium azide, 2 mM EGTA, 10 mM MgCl2, 1 mM DTT, pH 6.8) for 5 min at 37°C. The ATP hydrolysis reaction was then started by the addition of 5 mM ATP and it was allowed to proceed at 37°C for 40 min. After the incubation, SDS solution (0.1 mL of 5% SDS) was used to terminate the reaction. The liberation of inorganic phosphate was quantified by comparing the absorbance to a phosphate standard curve in a colorimetric assay [38].

### Immunohistochemical staining

The paraffin-embedded xenograft tumor tissue blocks were sectioned in 4 mm slices and placed on Anti slides. After de-waxing and hydration, the slides were rinsed in phosphate-buffered saline (PBS) and blocked for 10 min with 3% hydrogen peroxide to deprive the endogenous peroxidase activity. After antigen retrieval with the use of a microwave, the specimens were incubated with the anti ABCG2 mAb (diluted 1:1000 in PBS) overnight at 4°C. After washing with PBS, the sections were incubated with the secondary antibodies followed by fast staining with diaminobenzidine (DAB) according to the manufacturer's instructions (Dako Envision + Dual Link System-HRP detection kit). In negative controls, the primary antibody was replaced with PBS. The remaining procedures were performed in parallel with other specimens. Each slide was scored in a blinded fashion by two pathologists according to the manufacturer's recommended criteria at × 40 and × 200 magnification. Five visual fields for each immunostained section were examined randomly and recorded the ABCG2 staining index which defined by percent of positively stained tumor cells ( score 0, 1, 2, 3, 4) × staining intensity (score 0, 1, 2 and 3). The rate of positive cells was divided into less than 5% (score 0), 6% to 25% (score 1), 26% to 50% (score 2), 51% to 80% (score 3), and more than 80% (score 4). The staining intensity can be divided into three grades: no staining (score 0), slightly yellowish (score 1), brownish yellow (score 2), and dark brown (score 3).

### Western blotting

For Western blot analysis, protein concentrations were determined using the BCA Protein Assay (Thermo Fisher Scientific, Waltham, MA). Equal amounts of proteins were resolved on 10% sodium dodecyl sulfate-polyacrylamide gel electrophoresis (SDS-PAGE) gel and transferred to nitrocellulose membranes followed by blocking with 5% skimmed milk. Membranes were sequentially incubated with the primary and secondary antibodies. The protein bands were visualized using the enhanced chemiluminescence solutions and exposed to a Kodak medical x-ray processor (Kodak, Rochester, NY, USA).

### Relative levels of mRNA by real-time RT-PCR

Total RNA was extracted from different experi-mental group cells using Trizol reagent (Invitrogen, USA) according to the standard protocol. One microgram of total RNA was used for RT reaction in 20 μL of reaction volume, using a reverse transcription system (Thermo Fisher Scientific Inc., USA). SYBR Green Assay kit was used for real time PCR reaction, following manufacturer's protocol. The specific primers were described previously [39]. Data were analyzed using the 2^−ΔΔCT^ method and normalized by GAPDH expression in each sample.

### Statistical analysis

All experiments were repeated at least three times and representative results are presented. All data were shown as means ± SD. Statistical analyses were performed using the SPSS statistical software (SPSS 16.0). Any significant differences among mean values were evaluated by the Student's t-test. A two-sided *p* < 0.05 was accepted as statistical significance.
